# Identification of ZBTB26 as a Novel Risk Factor for Congenital Hypothyroidism

**DOI:** 10.3390/genes12121862

**Published:** 2021-11-24

**Authors:** Philipp Vick, Birgit Eberle, Daniela Choukair, Birgit Weiss, Ralph Roeth, Isabelle Schneider, Nagarajan Paramasivam, Markus Bettendorf, Gudrun A. Rappold

**Affiliations:** 1 Department of Zoology, University of Hohenheim, 70599 Stuttgart, Germany; philipp.vick@uni-hohenheim.de (P.V.); isabelle.schneider@uni-hohenheim.de (I.S.); 2 Department of Human Molecular Genetics, Institute of Human Genetics, Ruprecht-Karls-University Heidelberg, 69120 Heidelberg, Germany; Birgit.Koertje@web.de (B.E.); birgit.weiss@med.uni-heidelberg.de (B.W.); ralph.roeth@med.uni-heidelberg.de (R.R.); 3 Division of Paediatric Endocrinology, Children’s Hospital, Ruprecht-Karls-University Heidelberg, 69120 Heidelberg, Germany; daniela.choukair@med.uni-heidelberg.de (D.C.); markus.bettendorff@med.uni-heidelberg.de (M.B.); 4 Computational Oncology Group, Molecular Diagnostics Program at the National Center for Tumor Diseases (NCT) and DKFZ (German Cancer Research Center), 69120 Heidelberg, Germany; n.paramasivam@dkfz.de

**Keywords:** congenital hypothyroidism, thyroid anlagen, *Xenopus laevis*, thyroid dysgenesis, *ZBTB26*, *PAX8*

## Abstract

Congenital primary hypothyroidism (CH; OMIM 218700) is characterized by an impaired thyroid development, or dyshormonogenesis, and can lead to intellectual disability and growth retardation if untreated. Most of the children with congenital hypothyroidism present thyroid dysgenesis, a developmental anomaly of the thyroid. Various genes have been associated with thyroid dysgenesis, but all known genes together can only explain a small number of cases. To identify novel genetic causes for congenital hypothyroidism, we performed trio whole-exome sequencing in an affected newborn and his unaffected parents. A predicted damaging de novo missense mutation was identified in the *ZBTB26* gene (Zinc Finger A and BTB Domain containing 26). An additional cohort screening of 156 individuals with congenital thyroid dysgenesis identified two additional *ZBTB26* gene variants of unknown significance. To study the underlying disease mechanism, morpholino knock-down of *zbtb26* in *Xenopus laevis* was carried out, which demonstrated significantly smaller thyroid anlagen in knock-down animals at tadpole stage. Marker genes expressed in thyroid tissue precursors also indicated a specific reduction in the *Xenopus* ortholog of human Paired-Box-Protein PAX8, a transcription factor required for thyroid development, which could be rescued by adding zbtb26. Pathway and network analysis indicated network links of *ZBTB26* to *PAX8* and other genes involved in thyroid genesis and function. GWAS associations of ZBTB26 were found with height. Together, our study added a novel genetic risk factor to the list of genes underlying congenital primary hypothyroidism and provides additional support that de novo mutations, together with inherited variants, might contribute to the genetic susceptibility to CH.

## 1. Introduction

Congenital primary hypothyroidism (CH; OMIM 218700) is the most common metabolic disorder in newborns with an estimated prevalence of 1 in 2500–3500 births [1,2]. It is characterized by an impaired neurodevelopment and can lead to intellectual disability and growth retardation if untreated. Early diagnosis and therapy are absolutely critical to avoid brain damage. If the serum fT4 concentration is below, and TSH clearly above, the age-specific reference interval, then levothyroxine (LT4) treatment should be started immediately [3].

A genetic origin of CH has been supported by various evidence ([4]; recently reviewed by [2,5]). Familial forms of CH are, however, uncommon. Congenital hypothyroidism can be caused by thyroid dyshormonogenesis or thyroid dysgenesis. Only a minority of children with congenital hypothyroidism present thyroid dyshormonogenesis, while the majority (over 80%) present thyroid dysgenesis, a developmental anomaly of the thyroid. In thyroid dysgenesis, different developmental genes including *PAX8*, *FOXE1, NKX2-1, NKX2-5*, *HHEX*, and others have been shown to be affected and heterozygous dominant mutations prevail. All known genes together can only explain a low proportion of the cases [5].

To identify novel genetic causes for congenital hypothyroidism, we performed trio whole-exome sequencing in an affected newborn with high TSH levels together with his unaffected parents. A de novo missense mutation was identified in the *ZBTB26* gene (Zinc Finger A and BTB Domain containing 26). Two further *ZBTB26* gene variants were identified in a cohort of 156 individuals with congenital hypothyroidism. Pathway and network analysis indicated functional links of *ZBTB26* to other known genes underlying thyroid dysgenesis. GWAS studies indicated a significant association with height. Additionally, we report that morpholino knock-down of *zbtb26* in *X. laevis* led to smaller thyroid anlagen and that the *Xenopus* ortholog of human *PAX8* could be rescued by *zbtb26*, thus giving functional support for the involvement of thyroid dysgenesis.

## 2. Materials and Methods

### 2.1. Exome Sequencing and Filtering

Sequencing was performed using the Illumina HiSeq 2000 system. Raw sequence data were mapped to the 1000 genomes phase II reference genome (GRCh37 version hs37d5) using BWA 0.6.2. Samples showed an average coverage of 94.92×, and >99% of bases in the autosomal chromosomes of the target regions have ≥ 10× coverage and ≥20 QUAL score. SNVs and indels with a read depth of at least 10× and minimum QUAL score of 20 were considered. Non-synonymous and splice site affecting SNVs and all exonic indels were filtered further. The genotype predicted by Platypus was used to assign genotypes to the variants. Minor allele frequency (MAF) from gnomAD genes and exomes was used and variants with MAF > 0.1% were removed. In total, 1198 exomes and 3910 WGS samples from the in-house database were used as control to remove common variants and variants present in more than 1% of the control samples.

### 2.2. Sanger Sequencing of Variants

Thirty-three variants were confirmed by Sanger sequencing. Polymerase chain reaction (PCR) with primers indicated in Supplementary Table S1 was performed with HotStar Taq Polymerase and products analyzed on agarose gels and subsequently sequenced using an ABI machine (Genewiz).

### 2.3. Network and Pathway Analysis by Ingenuity Software

QIAGEN Ingenuity Pathway Analysis (IPA; https://digitalinsights.qiagen.com/, accessed on 15 September 2021) was applied to predict functional connections, and their interpretation was carried out in the context of protein networks that comprise protein–protein interactions and related biological functions as well as canonical signaling pathways.

### 2.4. Databases

TGP (https://browser.1000genomes.org); GnomAD (https://gnomad.broadinstitute.org/, accessed on 15 September 2021); CADD score (https://cadd.gs.washington.edu, accessed on 15 September 2021); dbSNP (https://www.ncbi.nlm.nih.gov, accessed on 15 September 2021); GTEx database (www.gtexportal.org, accessed on 15 September 2021); PROVEAN/SIFT (http://provean.jcvi.org/index.php, accessed on 15 September 2021); Polyphen2 (http://genetics.bwh.harvard.edu/pph2/, accessed on 15 September 2021); Mutation Taster (http://www.mutationtaster.org/, accessed on 15 September 2021); GWAS (www.ebi.ac.uk, accessed on 15 September 2021); Disease knowledge portal (https://cvd.hugeamp.org, accessed on 15 September 2021); IPA Ingenuity Systems (https://digitalinsights.qiagen.com/, accessed on 15 September 2021); and NIH Genotype-Tissue Expression (GTEx) project. Gene expression of ZBTB26 was accessed in 51 tissues by the NIH Genotype-Tissue Expression (GTEx) database, which is based on RNA-seq data from 8555 tissue samples obtained from 570 adult post-mortem individuals (V6, October 2015).

### 2.5. Experimental Approaches with X. laevis

*X. laevis* care and maintenance, morpholino design, and microinjections, as well as preparation of embryo sections, are given in detail in the Supplementary data.

### 2.6. In Situ Hybridization

A full-length zbtb26 RNA probe was used to perform whole-mount in situ hybridization (ISH). Fixation of embryos was done in MEMFA for 2–3 h at room temperature. ISH was performed with a *Xenopus* standard protocol [6]. RNA in situ probes were synthesized with SP6 or T7 polymerases. For tissue-specific analyses of mRNA enrichment after ISH, stained embryos were embedded in 4% formaldehyde for 3 h at room temperature. Embryos were embedded in a glutaraldehyde-crosslinked gelatin–albumin mix. Then sectioning was performed with a vibratome (Leica VT1000S; sections 30 µm).

### 2.7. Analyses of Thyroid Anlagen

Thyroid sizes were quantified at stage 45, when bilateral anlagen were still found in the ventral mesenchyme. Embryos were frontally sectioned and manually screened for thyroids. Equal settings were used to take pictures (10× objective of the same microscope, same filter set, same field of view, and same contrast and brightness adjustments). Embryos were only used when their anlagen (or the expected area with remnant tissue) were identified clearly. For individual embryos, the sections with the largest cross-sectional area of the thyroid anlagen were used for each slide [7]. The relative size of both thyroids was determined by measuring the surface area (square pixels) with the ImageJ polygon tool [8]. Then the obtained values were graphically represented as boxplots. Statistical calculations were performed using statistical R, with an unpaired Wilcoxon rank sum test with continuity correction (www.R-project.org, accessed on 15 September 2021).

## 3. Results

### 3.1. Clinical Data

Patients included in this study were identified by the newborn screening at the University Children’s Hospital Heidelberg. Screening of TSH concentration was measured in dry-blood spots and was carried out by the University of Heidelberg neonatal screening laboratory. Informed consent for exome or targeted sequencing was provided by the respective parents.

Patient 1 with a de novo L75S mutation in *ZBTB26* is a eutrophic newborn Caucasian male with 3390 g birth weight, 56 cm birth length, and 37 cm head circumference. Congenital hypothyroidism was detected with a TSH level of 179 mU/L and normal values for T4, T3, and fT4. Due to the dramatically elevated TSH level, treatment was initiated. Therapy was started with levothyroxine at day 6 and normalization of TSH level was achieved at day 7. No thyroid autoantibodies were detected, and thyroglobulin was initially 159 ng/mL, indicating remaining thyroid tissue. Postnatal ultrasound detected thyroid tissue in the expected position, but no associated malformations. Ultrasound at the age of 9 years, however, detected no thyroid tissues and thyroglobulin was 8.7 ng/mL while TSH was elevated, indicating thyroid hypoplasia. The boy presented normal intelligence, but dyscalculia and dyslexia at school age. To maintain euthyroidism, increasing dosage of levothyroxine was necessary over the time. The family members were Caucasian and non-consanguineous. The grandfather suffered from thyroid nodules, but the younger sister was healthy with normal thyroid function.

Patient 2 with H236R mutation is a eutrophic newborn Caucasian female. Congenital hypothyroidism was detected with a TSH level of 59 mU/L and reduced values for T4, T3, and fT4. Therapy was started postnatally with levothyroxine, and normalization of TSH level was achieved. The parents reported that they were healthy, but no thyroid examination was performed and they were not available for DNA analysis.

Patient 3 with an intronic variant is a eutrophic Caucasian newborn male, born at 38 gestational weeks with 3500 g birth weight and 53 cm birth length. Congenital hypothyroidism was detected with a TSH level >50 mU/L and reduced values for T4, T3, and fT4. Therapy was started postnatally with levothyroxine and normalization of TSH level was achieved. Ultrasound at the age of 6 years detected absence of both thyroid and ectopic tissues. There was normal psychomotoric development. To maintain euthyroidism, increasing dosage was necessary over the time. Healthy parents were not available for analysis.

Another patient 332,756 was identified by DECIPHER and presented a 418 kb deletion encompassing *ZBTB26* and 5 additional genes (*RC3H2, ZBTB26, RABGAP1, GPR21*, and *STRBP*). The patient had a pulmonary stenosis and hypothyroidism, as well as delayed speech and language development. This patient was not included in the analysis as the deleted region contained ZBTB26 as well as an additional 5 genes. Causality of ZBTB could therefore not shown.

### 3.2. Exome Sequencing, Filtering and Selection of Candidate Genes

To identify novel candidate genes implicated in the etiology of congenital hypothyroidism, exome sequencing has been carried out in an affected child and his unaffected parents. Raw sequence data were mapped to the 1000 genomes phase II reference genome (hs37d5) followed by a bioinformatical variant detection pipeline (see Material and Methods). After specific filtering, 650 candidate genes (SNV or Indels) were identified. Further filtering excluded those genes with a minor allele frequency above 0.01 in gnomAD, a consensus score above 1 and a negative intolerance score. Variants were then analyzed for their effect on protein function if they had been predicted as damaging by at least one of the prediction tools (Mutation Taster, Polyphen2, PROVEAN, and SIFT), and with a CADD above 13. A CADD score above 13 indicates that the variant is within the 5% of the most deleterious substitutions in the human genome. De novo variants were prioritized.

**Figure 1:**
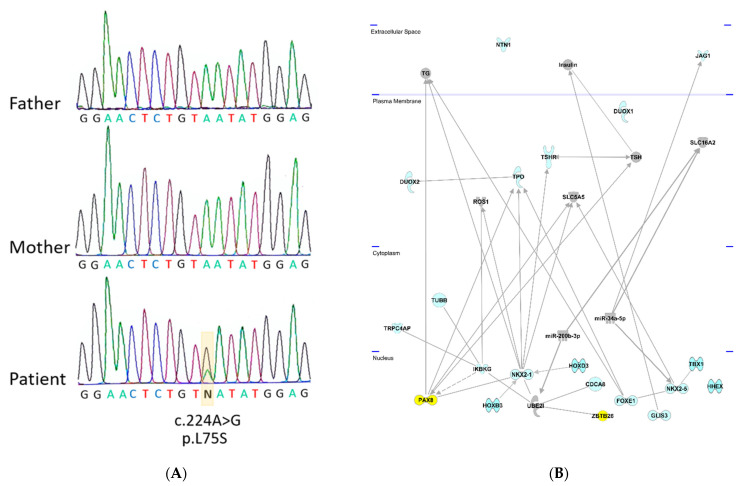
Identified variants in the *ZBTB26* gene and IPA Network analysis with known CH genes. (**A**) Sequence of trio with unaffected parents and affected child. Sanger sequencing in index case demonstrates a de novo variant c.224A>G in the *ZBTB26* gene which was not present in the unaffected parents. (**B**) Ingenuity (IPA) Network analysis. Known genes from the literature (in blue color) underlying congenital primary hypothyroidism were analyzed using the IPA network analysis. The newly identified *ZBTB26* gene was added to predict functional connections in the context of known protein networks. ZBTB26 and PAX8, both located in the nucleus, are highlighted in yellow. They are connected via NKX2-1 and UBEI. BOREALIN is also called CDCA8.

A de novo variant c.224A>G with the top CADD score of 25.4 was identified in the *ZBTB26* gene, leading to the amino acid exchange p.L75S (Figure 1A). ZBTB26 (Zink finger and BTB domain -containing protein 26) is a DNA-binding protein regulating transcription. It is a small, highly conserved gene with a transcript conservation between human and *Xenopus* of 73% identity. Somatic mutations in *ZBTB26* have been identified in different cancers including thyroid carcinoma (https://cancer.sanger.ac.uk/cosmic/gene/analysis?ln=ZBTB26, accessed on 15 September 2021). *ZBTB26* consists of two exons (the first exon is not translated) and a transcript length of 4455 base pairs, encoding a protein of 441 amino acids. The affected Leucine (L) in this individual is highly conserved in mice (*M. musculus*), rats (*R. norvegicus*), dogs (*Canis*), cows (*B. taurus*), chickens (*G. gallus*), and frogs (*Xenopus*), suggesting functional significance. The amino acid change was predicted to be disease-causing/damaging by all the used prediction programs and had previously not been identified in gnomAD (Supplementary Table S1).

### 3.3. Cohort Screening of 156 Children with Congenital Hypothyroidism for ZBTB26 Variants

To find out how frequent variants in the ZBTB26 gene are in children with primary hypothyroidism, Sanger Sequence analysis was carried out in an additional cohort of 156 patients with congenital hypothyroidism due to thyroid dysgenesis. Variants in two further individuals were identified, one with a c.707T>C substitution, causing an amino acid exchange p.H236R, and another with an intronic variant, 58 base pairs before the exon-intron junction. DNA of the parents of both patients was not available for analysis. Both variants were predicted to have a CADD score of 17.8 and 17.6. The amino acid change affected a highly conserved amino acid (Histidine 236) between humans and mice (*M. musculus*), rats (*R. norvegicus*), dogs (*Canis*), cows (*B. taurus*) and chickens (*G. gallus*). In gnomAD, it was detected in 4 out of 141,406 Europeans, resulting in an allele frequency of 0.00003098. The variant was completely absent in East Asians, but present in 487 Africans, indicating differences among populations. In summary, 2 out of 156 individuals with congenital hypothyroidism presented a risk variant of unknown functional significance in the *ZBTB26* gene (Supplementary Table S1).

### 3.4. Network Analysis

To find out if *ZBTB26* shares any common functional links with the known genes involved in primary hypothyroidism (indicated in blue color on Figure 1), Ingenuity pathway analysis was carried out. Network links between ZBTB26, UBE2I, NKX2-1, and PAX8 could be revealed; all reside in the nucleus (Figure 1B). Protein–protein interactions were identified between ZBTB26 and UBE2I, as well as UBE2I and NKX2-1 [9], and an interaction between PAX8 and NKX2-1 has been described [10]. Together, these data highlight a connection between *ZBTB26* and several known CH genes (Figure 1B).

### 3.5. GWAS and Disease Knowledge Databases

To predict the pathological relevance of the identified *ZBTB26* gene in a human phenotype, we consulted the Disease Knowledge Portal (https://cvd.hugeamp.org, accessed on 15 September 2021) and found GWAS associations of genome-wide significance of several intron variants of *CYP26B26* with height (9:125680547_C/T [*p*-value: 5.83 × 10^−14^]; 9:125683121_T/A [*p*-value: 4.45 × 10^−13^]; 9:125689694_C/T [*p*-value: 1.96 × 10^−12^]; and 9:125691119_T/A [*p*-value: 4.22 × 10^−12^]), strongly suggesting that this gene plays a role in final body height. In addition, a genome-wide association was also found with birth weight (*p*-value: 6.0 × 10^−16^) [11].

### 3.6. Experimental Data in X. laevis Provides Functional Support

**Figure 2:**
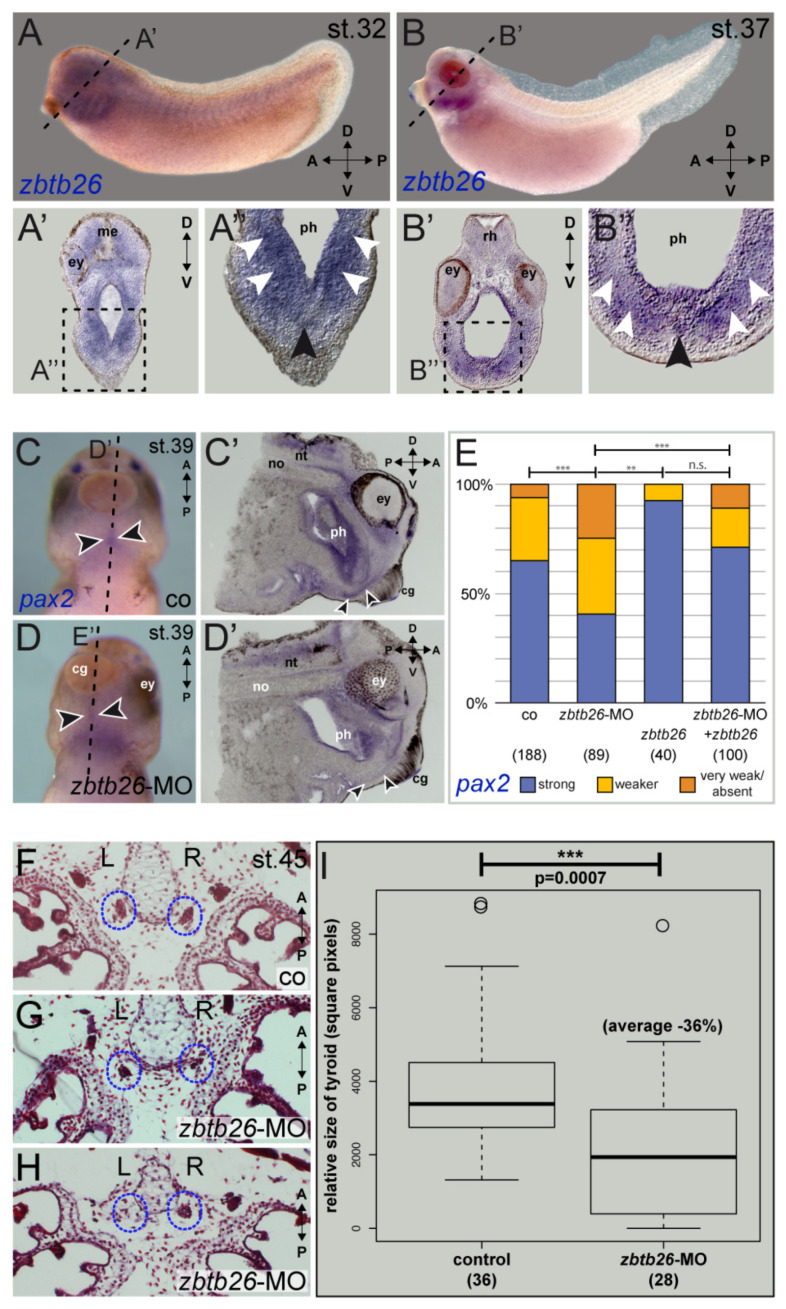
*zbtb26* has a conserved role in amphibian thyroid development. (**A**,**B**) Developmental expression of *zbtb26* mRNA in the thyroid anlage of the frog *X. laevis*. Transcripts in the head are detected ventrally in the medial ((**A**,**B’’**), black arrowheads) and ventro-lateral pharyngeal mesenchyme (white arrowheads) of stage 32 (**A**,**A’’**) and stage 37 (**B**,**B’’**). Plane of sections in (**A’**,**B’**) are indicated by dashed lines in (**A**,**B**); magnified areas in (**A’’**,**B’’**) are indicated by dashed boxes in (**A’**,**B’**). (**C**–**E**) *pax2* expression in the thyroid anlage of stage 39 embryos is reduced by *zbtb26* knockdown. Thyroid anlage is highlighted by arrowheads. *zbtb26*-MO-injected specimens showed reduced or lost expression of *pax2* in the thyroid (**D**,**D’**,**E**) compared to control embryos (**C**,**C’**,**E**). This could be rescued by co-injection of full-length *zbtb26* mRNA (**E**). Please note apparently more robust *pax2*-expression in zbtb26 mRNA-alone injected specimens (**E**). (**F**–**I**) Loss of *zbtb26* negatively affects development of the thyroid. Examples of paraffin-sections of control embryo (**F**) or specimens after knockdown of zbtb26 (**G**,**H**) with paired early thyroids highlighted by blue dotted circles. Please note the strongly reduced right thyroid anlage in (**G**) and the lost left anlage in (**H**). Dot plot of quantification of single thyroids showing significantly reduced average size in morphants versus controls (**I**). A, anterior; cg, cement gland; co, control; D, dorsal; ey, eye; me, mesencephalon; no, notochord; n.s., not significant; nt, neural tube; ph, pharynx; P, posterior; rh, rhombencephalon, R, right; st., stage; and V, ventral. Sample numbers indicated in brackets (in (**E**), embryos; in (**I**), left or right side of thyroid anlage). ** *p* < 0.01, *** *p* < 0.001.

As *ZBTB26* is expressed in human thyroid gland according to GTEx (among other tissues), we functionally assessed *ZBTB26* in thyroid development using the African clawed frog *X. laevis* as an animal model. Expression of the orthologous zbtb26 gene in the thyroid anlage was shown by in situ hybridization analyses at different stages of tadpole development (tailbud stage 32 to tadpole stage 45). Strong signals in the ventral foregut area (Figure 2A,B) and in the thyroid precursor were detected (Figure 2A’,B’). This was also the case in older tadpole stages, when thyroid anlagen are already split and relocated medially, as well as in the mouth floor epithelium (Figure S1A,B).

Morpholino-mediated knockdown of zbtb26 in the mesendodermal lineage of 4-cell stage embryos resulted in a strongly reduced thyroid anlage. We then analyzed expression of known *Xenopus* orthologs of known CH genes: forkhead box E3 (*foxe3*), hematopoietically expressed homeobox (*hhex*), NK2 homeobox 1 (*nkx2.1*), and paired box 2 (*pax2*), the amphibian functional ortholog of the mammalian *Pax8* gene [12,13,14,15,16,17]. Our experimental data show that *pax2* was significantly reduced or lost in the ventral thyroid anlagen of zbtb26 morphant embryos (Figure 2C–E), while *foxe3, nkx2.1*, and *hhex* expression was left unaltered (Figure S1C), suggesting that zbtb26 displays a regulatory role on *pax2.*

Mid-sagittal sections showed specific reduction in *pax2* in the pre-thyroid tissues of *Zbtb26* knockdowns compared to control siblings (Figure 2C’,D’). Further supporting a functional interaction of both genes, sections through the thyroid anlagen of a stage 37 embryo revealed overlapping expression in this area (Figure S1D,E). The effect on *pax2* was specific, as reintroduction of wildtype *Xenopus zbtb26* lacking the MO-binding sequence in the 5′UTR rescued the *pax2* expression in a significant manner. Interestingly, injecting *zbtb26* mRNA alone seemed to have an enhancing or stabilizing effect on *pax2* expression as well (Figure 2E).

Next, we wanted to analyze if this reduction in *pax2* expression after *zbtb26* loss of function also correlates with a change in thyroid tissue development in early tadpoles. We performed knockdown experiments, either targeting both anlagen or injecting unilaterally into the right or left lineage to hit only one half of the embryo (strictly separated in *Xenopus* early development), then used the opposite halves as internal controls. When analyzing tadpole frontal sections through the already separated thyroid anlagen at stage 45, we found most morphant thyroids to be reduced in size or not developed at all (Figure 2F–H). Quantification of bilaterally injected specimens revealed an average reduction in size by 36% in morphant embryos (Figure 2I). Similarly, specimens targeted unilaterally with the *zbtb26*-MO showed about 28% smaller thyroid anlagen on the injected side than those of the non-injected endogenous control halves (Figure S1F). These results support the conclusion that zbtb26 expressed in the thyroid precursor tissue is required for normal thyroid development.

## 4. Discussion

Since the identification of the first causal gene for thyroid dysgenesis PAX8 in 1998 [18], a number of genes underlying congenital primary hypothyroidism (CH) have been reported to play a role in this phenotype (e.g., *TSHR, NKX2-1, NKX2-5, FOXE1, GLIS3, JAG1, TBX1, NTN1, CDCA8, HOXD3, HOXB3, CDCH8, TUBB1*, and *TRPC4AP*) [5,19,20]. These advances have contributed to our understanding of thyroid development and provided very useful information for genetic diagnosis. However, the underlying molecular mechanisms are still poorly understood, and the vast majority of patients and their families are still lacking a genetic diagnosis.

We have provided genetic and functional evidence that *ZBTB26* is a further risk gene for this disorder. The *ZBTB26* gene is a member of the large ZBTB family of proteins, which are highly conserved transcription factors with essential functions during development (Cheng et al., 2021). All ZBTB family members contain a BTB protein binding and Kruppel-type zink finger DNA binding domain. The BTB domain is used for protein–protein interactions, while the zinc finger domain primarily functions as a DNA binding module. The precise role of most ZBTB family members is still unknown. Several ZBTB proteins have emerged as critical factors during development which regulate the lineage commitment, differentiation and function of lymphoid cells as well as many other developmental processes [21].

In this study, we have identified a de novo missense in *ZBTB26* detected in a child with CH, which was not present in his unaffected parents. This child, as well as the other patients analyzed, were all derived from the German newborn screening program. Two additional variants in *ZBTB26* were identified in the cohort of 156 children with CH, but as the parents were not available in this study, it remains unsolved if these variants occurred de novo or not. As the detected *ZBTB26* variants were rare in this cohort (2/156), a high genetic heterogeneity is presumed, which is in line with former investigations.

An oligogenic model of inheritance for CH has also been proposed by previous studies, possibly together with a modulation by environmental modifiers [5,22]. This may also apply to our patients, at least to the patient, where exon sequencing has been performed. In this patient with the de novo *ZBTB26* mutation, further weak variants in additional known CH genes (*PAX8, TUBB1*) were transmitted from either parent to the child (the *PAX8* variant from mother; the *TUBB1* variant from father), suggesting that a combination of different variants would be needed to lead to the full-blown phenotype (Supplementary Table S2). The co-occurrence of de novo and familiar mutations in genes involved in CH also highlight that epistasis between different specific gene loci can modulate the risk for CH. Different susceptibility variants for CH variants may thus act together, and in some cases, additional epigenetic or environmental factors may be also necessary for the emergence of the disorder. This oligogenic model of inheritance or multi-hit model for CH has been already proposed before [5,22] and may also apply to our patients.

Finally, functional studies have shown that *ZBTB26* represents a convincing risk gene for CH. Network analysis has shown that *ZBTB26* is linked to several known CH genes (*CDCA8, NKX2-1*, and *IKBKG*) via *UBE2I,* and according to the GTEX database, *ZBTB26* has one of its highest expressions in the thyroid. In *Xenopus*, which was used as an animal model, *zbtb26* transcripts were found in the early thyroid anlagen and continued to be active in early thyroids at different stages of tadpole development (Figure 2 and Figure S1A,B). Gene activity can easily be manipulated in *Xenopus* by microinjection into its large early embryos right after fertilization. These knockdown experiments demonstrated significantly smaller thyroid anlagen and smaller thyroids at the tadpole stage, thus indicating a direct effect of its reduced expression.

In summary, a causal link of *ZBTB26* variants and CH is supported by several lines of evidence: Variants with evolutionary conservation of affected amino acids and good algorithm predictions with an effect on protein function and low frequency in the general Caucasian population were detected in altogether 3/157 individuals with CH. In addition, reported associations with final height and the gene expression patterns in the thyroid in human and frog supported these findings. Finally, the regulatory role on pax2 expression, and the in vivo requirement for early thyroid organogenesis in tadpole furthermore speak for a relevance of this gene in CH. Our study also provides additional support that de novo mutations, together with inherited variants, might contribute to the genetic susceptibility to CH. To date, it is not clear how many genes contribute to the phenotype of CH and how these different variants interact with each other. A better knowledge of these complex interactions will be needed to fully understand this common endocrine disorder.

## Data Availability

All data can be obtained by the authors.

## Supplementary Materials

The following are available online at https://www.mdpi.com/article/10.3390/genes12121862/s1: Table S1: Identified ZBTB variants in patients with congenital hypothyroidism, Table S2: Additional variants in known hypothyroid genes and gnomAD analysis of variants compared with *ZBTB26*, Figure S1: *zbtb26* has a conserved role in amphibian thyroid development; Supplementary data: *Xenopus laevis:* care and maintenance, morpholino design, microinjection and embryo sections.
