# Supplementary material for: Identification of ZBTB26 as a Novel Risk Factor for Congenital Hypothyroidism
Source: Genes (Basel). 2021 Nov 24;12(12):1862. doi: 10.3390/genes12121862 (PMC8701029; doi:10.3390/genes12121862)
Supplement: Supplementary file 1 [file genes-12-01862-s001.zip › Suppl.Table S1.pdf]

Exome-sequenced Trio

Patient	Nucleotide Exchange	Amino acid Exchange	Location	Position	dbSNP ID	Mutation	Mutation taster	Polyphen2	Provean	SIFT	CADD
**1.**	c.224A>G	p.L75S	exonic	Chr9:125681990	-----	Missense *de novo*	disease causing	possibly damaging	deleterious	damaging	25.4

Variants in ZBTB26 in a congenital hypothyroidism cohort of 156 patients

Patient	Nucleotide Exchange	Amino acid Exchange	Location	Position	dbSNP ID	Mutation	Mutation taster	Polyphen2	Provean	SIFT	CADD
**2.**	c.707T>C	p.H236R	exonic	Chr9:125681507	rs7856488	Missense	disease causing	benign	neutral	tolerated	17.8
**3.**	C>G	-----	intronic	Chr9:125682281	-----	SNV	disease causing	-----	-----	-----	17.6

gnomAD analysis of identified ZBTB26 variants

Patient/ Gene	Amino acid Exchange	Allele frequency in European (Non-Finnish) in gnomAD	European (Non-Finnish) in gnomAD	Latino in gnomAD	African in gnomAD	East Asian in gnomAD	South Asian in gnomAD	Other in gnomAD	Total Allele Counts in gnomAD	Total number homozygous in gnomAD	Allele number in gnomAD
1. *ZBTB26*	p.L75S	-----	-----	-----	-----	-----	-----	-----	-----	-----	-----
2. *ZBTB26*	p.H236R	0.00003098	4	34	487	0	8	7	540	5	282812
3. *ZBTB26*	-----	-----	-----	-----	-----	-----	-----	-----	-----	-----	-----

Supplementary Table S1: Identified ZBTB variants in patients with congenital hypothyroidism
