# Supplementary material for: Identification of ZBTB26 as a Novel Risk Factor for Congenital Hypothyroidism
Source: Genes (Basel). 2021 Nov 24;12(12):1862. doi: 10.3390/genes12121862 (PMC8701029; doi:10.3390/genes12121862)
Supplement: Supplementary file 1 [file genes-12-01862-s001.zip › Suppl.Table S2.pdf]

Additional Variants in known Hypothyroidism genes

Gene	Nucleotide Exchange	Amino acid Exchange	Location	Position	dbSNP ID	Mutation	Parental origin	Mutation taster	Polyphen2	Provean	SIFT	CADD
PAX8	c.985A>G	p.F329L	exonic	Chr2:113993073	rs3188996	missense	mother	disease causing	possibly damaging	neutral	tolerated	24.7
TUBB1	c.406G>A	p.V136I	exonic	Chr20:57598888	rs62639976	missense	father	polymorphism	benign	neutral	damaging	18.2

gnomAD Analysis of Variants compared with *ZBTB26*

Gene	Amino acid Exchange	Allele frequency in European (Non-Finnish) in gnomAD	European (Non-Finnish) in gnomAD	Latino in gnomAD	African in gnomAD	East Asian in gnomAD	South Asian in gnomAD	Other in gnomAD	Total Allele Counts in gnomAD	Total number homozygous in gnomAD	Allele number in gnomAD
PAX8	p.F329L	0.02668	1278	95	48	2	133	37	1778	26	119182
TUBB1	p.V136I	0.00006212	3	0	1	2	0	0	6	0	120288
ZBTB26	p.L75S	-----	-----	-----	-----	-----	-----	-----	-----	-----	-----

Supplementary Table S2
