# Supplementary material for: Identification of ZBTB26 as a Novel Risk Factor for Congenital Hypothyroidism
Source: Genes (Basel). 2021 Nov 24;12(12):1862. doi: 10.3390/genes12121862 (PMC8701029; doi:10.3390/genes12121862)
Supplement: Supplementary file 1 [file genes-12-01862-s001.zip › supplementary Figure S1.pdf]

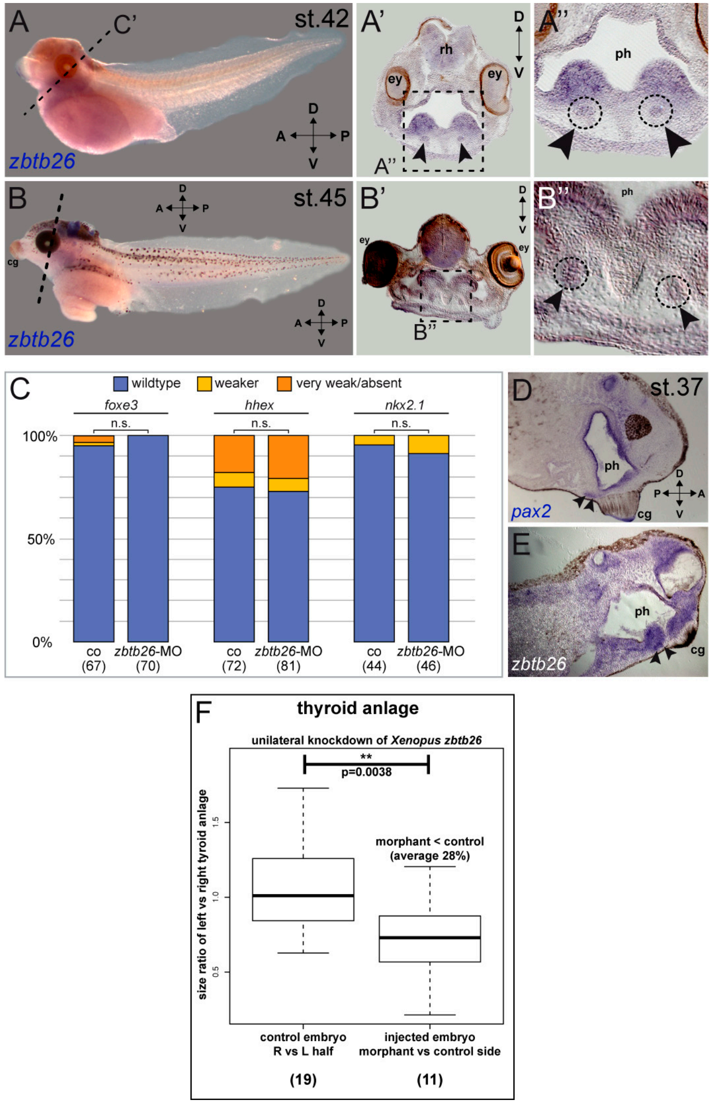

Figure S1. ***zbtb26*** **has a conserved role in amphibian thyroid development**

(A, B) Later expression of *zbtb26* mRNA in thyroid tissues of st. 42 (A) and st. 45 (B) *Xenopus* tadpoles.

Expression can still be found in the split lateral thyroid (A-B'', black arrowheads). Planes of section in A' and B' are indicated by dashed lines in A and B, respectively; magnified area in A'' and B'' is indicated by dashed boxes in A' and B', respectively; thyroid anlage highlighted by doted circles in A'' and B''.

(B-D) Other thyroid markers are not influenced by *zbtb26* knockdown.

Knockdown of *zbtb26* has no significant impact on the expression of *foxD3*, *hhex* or *nkx2.1* in the thyroid anlage (C). Mid-sagittal sections illustrating overlap of expression of *pax2* (D) and *zbtb26* (E) mRNA in the thyroid anlage of st. 37.

(F) Unilateral loss of *zbtb26* negatively affects development of the thyroid anlage in the morphant half of the embryos.

Dot plot of size ratios of the two thyroid anlagen of one embryo. Quantification shows significantly reduced size of morphant versus control side in unilaterally injected embryos (right) but not right versus left sides in control embryos (left).

A, anterior; cg, cement gland; co, control; d, dorsal; ey, eye; n.s., not significant; ph, pharynx; P, posterior; Rh, rhombencephalon; st., stage; V, ventral. Sample numbers indicated in brackets (number of embryos in C and F). *$p<0.05$, **$p<0.01$, ***$p<0.001$

Table S 2. Additional variants in known hypothyroidism genes

Supplementary data

***Xenopus laevis* care and maintenance**

Frogs were shipped from Nasco (901 Janesville Avenue, P.O. Box 901, Fort Atkinson, WI, USA). Handling, care and experimental manipulations of animals was approved by the Regional Government Stuttgart, Germany (V340/17 ZO and V349/18 ZO) according to German regulations and laws. Animals were kept at the appropriate conditions (pH=7.7, 20°C) at a 12 h light cycle in the animal facility. To induce ovulation, female frogs were injected subcutaneously with 300-700 units of human chorionic gonadotropin (hCG; Sigma), depending on weight and age. Only healthy clutches of embryos with normal survival rates were used for the experiments. Individual embryos from one batch were randomly picked and used either as control or tested specimens.

Morpholino design and microinjections in *Xenopus laevis*

A *zbtb26* 5′UTR-MO targeting both *X. laevis* homeologs was designed, the sequence is: 5′-CTTTGGCTACTCAAATTAATATGCA-3′. Embryos were injected at 4-cell stage into the dorsal blastomeres, either bilaterally or unilaterally left/right, targeting the future ventral foregut (i.e. including thyroid anlagen) using a Harvard Apparatus setup. Drop size was calibrated to 4-8 nl / injection, MO doses were 1-2 pmol / injection. Either non-injected littermates or non-injected halves of the same embryo served as (internal) controls.

Embryo sections for thyroid analyses in *Xenopus laevis*

Stage 45 embryos (about 4 days of development) were fixed in Bouin solution at room temperature. Sections for thyroid analyses were made using a microtome (Reichert, Austria; sections 10µm). Embryos were dehydrated in isopropyl alcohol and embedded in paraffin wax blocks. Paraffin sections were then stained with 'nuclear fast red' (Roth, Karlsruhe, Germany).
